# Immune system cells modulation in patients with reproductive issues: A systematic review approach

**DOI:** 10.5935/1518-0557.20230044

**Published:** 2024

**Authors:** Gabriel Acácio de Moura, Yasmim Mendes Rocha, Francisca Lariza Damascieira Moura, Janaína de Oliveira Freitas, João Pedro Viana Rodrigues, Vanessa Pinheiro Gonçalves, Roberto Nicolete

**Affiliations:** 1 Post-Graduate Program in Pharmaceutical Sciences (PPGCF) Federal University of Ceará (UFC), Fortaleza, CE, Brazil; 2 Oswaldo Cruz Foundation (FIOCRUZ CEARÁ), Eusébio, CE, Brazil; 3 Ceará Hematology and Hemotherapy Center (HEMOCE), Fortaleza, CE, Brazil; 4 North Northeast Biotechnology Network (RENORBIO), State University of Ceará (UECE), Fortaleza, CE, Brazil

**Keywords:** abortion, embryo implantation, assisted reproductive techniques, cytokines

## Abstract

The aim of this study was to carry out a systematic literature review to investigate the main immune cells responsible for implantation failures. We selected papers from PubMed, Embase and Virtual Health Library databases. Eligible articles included publications between January 1, 2010 and April 24, 2022. Inclusion criteria were: observational and case-control studies; and the exclusion criteria were: review papers, letters to the editor, abstracts, animal studies and case reports. We extracted the following information: day of collection, number of patients, control group, age of patients, type of sample used, immune cells and cytokines. As main findings in our mapping, we found that in peripheral blood, CD3+, CD4+, CD8+, CD16+, CD56+, CD57+, CD69+, CD154+, CD158a+, NKp46 cells were increased and the CD4+, CD45+, Foxp3 and NKp46 markers were reduced. From the endometrial biopsies, there was an increase in CD3+, CD4+, CD5+, CD8+, CD16+, CD25+, CD45+, CD56+, CD57+, CD68+, CD127+ and a reduction in CD45+, CD56+, NKp46 and FoxP3 cells. Cytokines found increased in peripheral blood included IL-6, IL-10, IL-17, INF-γ, TGF-ß, TNF-α; while IL-4, IL-6, IL-10, IL-35, FoxP3, TGF-ß, SOCS3 were reduced. As for the biopsies, there was an increase in IL-2, IL-6, IL-17, IL-22, IL-23, INF-A1, INF-B1, INF-γ, TNF-R and a reduction in IL-6, IL-10, INF-γ, TGFß, TNF-α. We concluded that immune cells can be modulated during pregnancy failure, but further studies are needed to elucidate the modulating effect of the immune system on the endometrium of these patients.

## INTRODUCTION

Assisted Reproductive Techniques (ART) have evolved exponentially to improve the rates of obtaining clinical pregnancy in couples with infertility. Despite these advances, embryo implantation is still a limiting step in the success rate of these ARTs (van Hoogenhuijze *et al*., 2017). Globally, the in vitro fertilization (IVF) technique enables a rate of 25 to 30% live births per initiated cycle, differing from rodents or rabbits, which have a 95% implantation rates. This fact may be directly associated with endometrial and decidual control in embryo implantation events in different mammalian species (Simon *et al*., 2020). Several factors can interfere with the embryo implantation process, such as endometrium, embryo, anatomical structure, lifestyle, thrombophilic conditions, and immunological factors (Kalem *et al*., 2020).

Among these mechanisms, the immunological factor stands out. During the communication between the endometrium and the embryo, a pattern of pro-inflammatory cytokines is established to help regulate the endometrium receptivity (Madkour *et al*., 2016). We assume that the uterus local immune system (IS) significantly assists in tolerance to the semi-allogeneic graft of the conceptum (Sho *et al*., 2017). Furthermore, it has been proposed that IS dysfunction in the endometrial milieu is related to mechanisms that compromise embryo implantation, potentially causing problems such as Recurrent Implantation Failure (RIF) and increased Recurrent Abortion Rates (RAR) in ART procedures (Singh *et al*., 2019). Given the potential relevance of the IS in embryo implantation rates, it may become an excellent reliable, non-invasive biomarker for verifying gestational success rates (Qasemi *et al*., 2021).

Despite multiple improvements in ARTs, implantation failure is still considered a relevant issue (van Hoogenhuijze *et al*., 2017). Such a fact is mainly due to the emotional burden that unsuccessful ART cycles can generate in patients undergoing these procedures (Bashiri *et al*., 2018). Implementing biomarkers in reproductive medicine may be a viable alternative for reducing implantation problems (Palmer & Barnhat, 2013). As an element distributed throughout the body, the IS may become an effective biomarker for specific implantation events (Qasemi *et al*., 2021). Thus, our study aims to investigate, through a systematic literature review, the main immune cells present in the process of embryo implantation failure.

## MATERIALS AND METHODS

### Study

This study is a systematic literature review based on papers from PubMed, Embase, and Virtual Health Library (VHL) databases. To this end, the study was prepared following the guidelines for Reporting Items for Systematic Reviews and Meta-Analysis (PRISMA). It is worth noting that the entire protocol of this review was submitted to the National Institute for Health Research database (CRD42022343288).

### Eligibility Criteria

For study selection, we adapted the PICOS methodological model used by Rocha *et al*. (2021). The full summary description of the criteria is presented in Table 1. The eligibility criteria adopted were:

**Table 1:** Systematized search using the PICOS method.

Description	Abbreviation	Question Components
Population	P	Women who used ARTs had RAR, RIF, and IVF failure.
Intervention	I	Performance of blood collection or endometrial biopsy for dosage of immune cells and/or inflammatory cytokines before and/or after embryo transfer.
Comparison	C	Fertile women who achieved clinical pregnancy.
Outcome	O	Obtained key IS cells and/or secreted cytokines found in peripheral blood or from endometrial cells.
Study	S	Observational and case-control studies.

ART: Assisted Reproductive Techniques; IVF: In vitro fertilization; RAR: Recurrent Pregnancy Loss; RIF: Recurrent Implantation Failure; IS: Immune System.

1. **Studies Included:** Clinical and case-control trials that addressed the proposed topic;
2. **Publication Period:** The selected studies were published between January 1, 2010, and April 24, 2022;
3. **Language of the Studies:** we analyzed papers in English, Portuguese (Brazil), and Spanish;
4. **Intervention Used:** Patients with RAR, RIF, and fertilization failures submitted to ART, who underwent before or after endometrial biopsy, treatments, or blood collection to study their immune profile; and
5. **Study outcome:** Obtaining the major IS cells and/or secreted cytokines found in peripheral blood or endometrial cells.

### Database Search

We searched the “PubMed”, “Embase” and VHL databases. The search was done manually using a combination of previously selected keywords from the Medical Subjects Headings (MESH) database in the advanced search. The following keywords were used: (immune system) AND (embryo implantation) AND (women).

### Inclusion and Exclusion Criteria

To filter the best results within the suggested scope of the review, we used the following inclusion and exclusion criteria:

Inclusion Criteria

1. Observational Studies; and
2. Case-Control Studies.

Exclusion Criteria

1. Articles out of scope;
2. Literature reviews;
3. Letter to the editor;
4. Abstracts;
5. Animal Studies; and
6. Case Reports.

### Methodological Quality Assessment of Studies

To reduce the chance of bias among the selected papers we assessed the risk of methodological bias. To do so, we used the Newcastle Ottawa scale for bias assessment of observational studies. Studies considered to have a low risk of bias entered our review assessment. All data from the methodological evaluation are presented in Table 2.

**Table 2:** Methodological Quality of Cross-sectional Studies (Newcastle Ottawa Scale).

Study	Newcastle Ottawa Scale-items Score								
	Criteria								
	Selection				Comparability 1a	Results			Total
	1	2	3	4		1	2	3	
Yang *et al*., 2010	1	1	0	1	1	1	1	1	7
Tuckerman *et al*., 2010	1	1	0	1	1	0	1	1	6
Karami *et al*., 2012	1	1	1	1	1	1	1	1	8
Zhou *et al*., 2012	1	1	1	1	1	1	1	1	8
Junovich *et al*., 2013	1	1	0	1	1	0	1	1	6
Ozkan *et al*., 2014	1	1	1	1	1	1	1	1	8
Liu *et al*., 2014	1	1	1	1	1	1	1	1	8
Saifi *et al*., 2014	1	1	1	1	1	1	1	1	8
Chernyshov *et al*., 2014	1	1	1	1	1	1	1	1	8
Santillán *et al*., 2015	1	1	1	1	1	1	1	1	8
Dons’koi, 2015	1	1	1	1	1	1	1	1	8
Galgani *et al*., 2015	1	1	1	1	1	1	1	1	8
Saifi *et al*., 2016	0	1	1	1	1	1	1	1	7
Wang *et al*., 2017	1	1	0	1	1	1	0	1	6
Yin *et al*., 2017	1	1	1	1	1	1	1	1	8
Jiang *et al*., 2017	1	1	0	1	1	1	0	1	6
Kuon *et al*., 2017	1	1	1	1	1	1	1	1	8
Fukui *et al*., 2017	1	1	0	1	1	1	0	1	6
Chen *et al*., 2017	1	1	1	1	1	1	1	1	8
Chen *et al*., 2018	1	1	1	1	1	1	1	1	8
Kolanska *et al*., 2019	1	1	0	1	1	1	0	1	6
Liu *et al*., 2019	1	1	1	1	1	1	1	1	8
Koushaeian *et al*., 2019	1	1	1	1	1	1	1	1	8
Donoghue *et al*., 2019	0	1	1	1	1	1	1	1	7
Zhao *et al*., 2020	0	1	1	1	1	1	1	1	7
Amjadi *et al*., 2020	0	1	1	1	1	1	0	1	6
Sauerbrunn-Cutler *et al*., 2021	0	1	1	1	1	1	1	1	7
Comins-Boo *et al*., 2021	1	1	1	1	1	1	1	1	8
Zhao *et al*., 2021	0	1	1	1	1	1	0	1	6
Kuroda *et al*., 2021	1	1	1	1	1	1	1	1	8
Dons’koi *et al*., 2021	1	1	0	1	1	1	0	1	6

Selection 1: Representativeness of the exposed cohort; Selection 2: Selection of the unexposed cohort; Selection 3: Exposure determination; Selection 4: Demonstration that the result of interest was not present in the baseline; Comparability 1a and 1b: Comparability of cohorts based on design or analysis; Results 1: Results assessment; Results 2: Follow-up of cohorts; Results 3: Adequacy of cohort follow-up.

### Data Collection and Extraction

After screening the articles mentioned above, they were evaluated by four authors (MGA; RYM; FLDM; JOF). In case of divergence, a fifth author (NR) specialized in the field would reevaluate the paper and the results were tabulated. The data extracted from each paper were:

1. Day on which the sample was collected;
2. Number of patients;
3. Control group;
4. Age of patients;
5. Type of sample used; and
6. Immune cell mediators.

## RESULTS

### Methodological Screening

**Figure 1:**
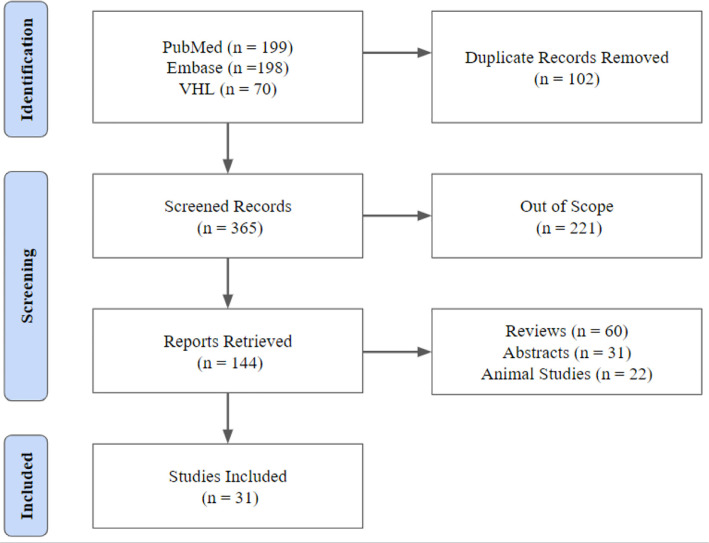
Methodological screening.

According to the methodological model used to formulate the review, we found 467 papers. Of these, only 31 were selected for the review. Overall, 1,800 patients with a mean age between 20 to 39.9 years were evaluated. The data are available in Table 1. It is worth pointing out that the primary sampling methodologies to identify immunological profiles of these patients were acquired from endometrial biopsies and peripheral blood sampling. Based on this, we extracted the data extraction from these two different methodologies. Figure 1 depicts the entire methodological screening.

### Immune Cell Mapping in Peripheral Blood

We found that in the peripheral blood of patients with implantation problems, there was an increase in cells compared to controls. The main increased cells were CD3+, CD4+, CD8+, CD16+, CD56+, CD57+, CD69+, CD154+, CD158a+, NKp46 (Yang *et al*., 2010; Karami *et al*., 2012; Chernyshov *et al*., 2014; Santillán *et al*., 2015; Dons’koi, 2015; Jiang *et al*., 2017; Dons’koi *et al*., 2021; Comins-Boo *et al*., 2021). On the other hand, CD4+, CD45+, Foxp3, and NKp46 cells were reduced in patients with reproductive issues, compared to control groups (Zhou *et al*., 2012; Fukui *et al*., 2017; Liu *et al*., 2019; Comins-Boo *et al*., 2021). Th1 type immune responses were also increased in two studies (Saifi *et al*., 2014; Kuroda *et al*., 2021).

### Immune Cell Assessment in Endometrial Biopsy Samples

The literature also infers that implantation issues may modulate the immune response in the endometrium. There was a marked increase of cells in samples from these patients when compared to control groups. Among these, CD3+, CD4+, CD5+, CD8+, CD16+, CD25+, CD45+, CD56+, CD57+, CD68+, CD127+ (Tuckerman *et al*., 2010; Junovich *et al*., 2013; Santillán *et al*., 2015; Galgani *et al*., 2015; Wang *et al*., 2017; Kuon *et al*., 2017; Jiang *et al*., 2017; Chen *et al*., 2017; 2018; Zhao *et al*., 2020; 2021; Sauerbrunn-Cutler *et al*., 2021). In contrast, cells such as CD45+, CD56+, NKp46 and Foxp3 had reduced levels in endometrial biopsies from patients with RIF, RAR, or who resorted to ART (Jiang *et al*., 2017; Fukui *et al*., 2017; Sauerbrunn-Cutler *et al*., 2021).

### Inflammatory cytokines in the peripheral blood of patients

In our review, there was an increase in inflammatory cytokines in the peripheral blood of patients with gestational complications, among which we list IL-6, IL-10, IL-17, INF-γ, TGF-ß, TNF-α (Ozkan et al., 2014; Saifi et al., 2014; Yin et al., 2017). In contrast, a group of inflammatory cytokines was reduced in patients with gestational complications in six studies, such as IL-4, IL-6, IL-10, IL- 35, FoxP3, TGF-ß, SOCS3 (Junovich et al., 2013; Zhou et al., 2012; Ozkan et al., 2014; Saifi et al., 2014; 2016; Koushaeian et al., 2019). All data are detailed in Table 3.

**Table 3:** Immunological profile of patients with peripheral blood collection.

Author	Collection Day	Number of Patients	Control Group	Age (Average)	Cytokine panel	Immune Cells
Yang *et al*., 2010	-	18 RIF group 17 RAR group	Fertile women	RIF (35.6 years) RAR (35.1 years)	No significant differences	↑ CD154+ ↑ CD69+
Karami *et al*., 2012	-	20 RIF group 23 RAR group	Fertile women	RIF (31.5 years) RAR (30 years)	-	↑ CD56+
Zhou *et al*., 2012	-	34 non-pregnant women	Pregnant	32.4 years	-	↓ CD4+; ↓ FoxP3
Junovich *et al*., 2013	After LH peak (5-9 days)	26 fertile group	Fertile women	-	↓ IL-6	No significant differences
Ozkan *et al*., 2014	Cycle (21 day)	80 infertile group	Fertile women	30.2 years	↓SOCS3;↓IL-35;↓IL-4; ↑ IFN-γ;↑ IL-17;↑ TGFβ; ↑ IL-6;↑ TNF-α;↑ IL-10	-
Saifi *et al*., 2014	Cycle (19-23 days)	20 RAR group	Fertile women	-	↑ IL-17;↓ TGFβ	-
Chernyshov *et al*., 2014	Cycle (17-23 days)	75 failure to conceive group 18 abortion group	Conception group	23-35 years	-	↑CD56+; ↑CD158a+; ↑CD4+; ↑CD8+
Santillán *et al*., 2015	After LH peak (5-9 days)	73 RIF group	Fertile women	-	-	↑ CD56+
Dons’koi, 2015	-	90 non-pregnant group	Conception group	28.2 years	-	↑ T CD3+ ↑ T CD8+
Saifi *et al*., 2016	Cycle (19-23 days)	20 RPL group	Fertile women	30.72 years	↓ IL-10	-
Jiang *et al*., 2017	After LH peak (7-9 days)	32 RIF group	Fertile women	32 years	-	↓ CD45+
Yin *et al*., 2017	Cycle (3-5 days)	56 RIF women	Fertile women	34,9 years	↑ IFN-γ;↑ TNF-α	No significant differences
Fukui *et al*., 2017	-	34 RIF group 28 RAR group	Fertile women	RIF (35.3 years) RAR (33.8 years)	-	↓ NKp46
Kolanska *et al*., 2019	-	41 RIF group 54 RAR group	Fertile women	RIF (36.3 years) RAR (36.3 years)	-	No significant differences
Liu *et al*., 2019	Luteal phase (7-9 days)	7 RIF group 20 RAR group	Fertile women	RIF (31.1 years) RAR (33.3 years)	-	No significant differences
Koushaeian *et al*., 2019	-	23 RIF group	Fertile women	39.9 years	↓ IL-10	No significant differences
Kuroda *et al*., 2021	-	79 RIF group 81 RAR group 40 infertile group	Fertile women	RIF (37.6 years) RAR (38 years) Infertile (35.4 years)	-	↑ Th1
Dons’koi *et al*., 2021	Implementation phase (16-20 days)	57 RIF group	Fertile women	29.5 years	-	↑ NKp46+
Comins-Boo *et al*., 2021	-	25 RIF group 58 RAR group	Fertile women	RIF (36.0 years) RAR (36.9 years)	-	↓ NKp46+

### Inflammatory cytokines from endometrial biopsies

Two studies reported an increase in inflammatory cytokines in endometrial biopsies, such as IL-2, IL-6, IL-17, IL-22, IL-23, INF-A1, INF-B1, INF-γ, TNF- R (Galgani et al., 2015; Amjadi et al., 2020). In endometrial tissues, some inflammatory cytokines had their levels reduced in four studies, such as IL-6, IL-10, INF-γ, TGFß, TNF-α (Junovich et al., 2013; Galgani et al., 2015; Wang et al., 2017; Fukui et al., 2017). All data are detailed in Table 4.

**Table 4:** Immunological profile of patients who underwent endometrial biopsy procedures.

Author	Collection Day	Patients (n)	Control Group	Age (Average)	Cytokine panel	Immune Cells
Tuckerman *et al*., 2010	After LH peak (7-9 days)	40 RIF group	Fertile women	35 years	-	↑ CD56+
Junovich *et al*., 2013	After LH peak (5-9 days)	26 infertile group	Fertile women	-	↓ IL-6	↑ CD16+
Liu *et al*., 2014	After LH peak (7-9 days)	72 RIF group 94 RAR group	Fertile women	RIF (33.4 years) RAR (33.1years)	-	No significant differences
Santillán *et al*., 2015	After LH peak (5-9 days)	73 RIF group	Fertile women	-	-	↑ CD56+
Galgani *et al*., 2015	Cycle (21-23 days)	15 RIF group 13 RAR group	Fertile women	RIF (34.5 years) RAR (33.6 years)	↑ TNF-R;↑IL-22;	↑ CD3+;↑ CD4+ ↑ CD5+’↑ CD8+ ↓ FoxP3+
Wang *et al*., 2017	+14 TE	53 pregnant group	Pregnant women	33.1 years	↓ IL-10; ↓ TGF-β	↑ CD4+;↑ CD25+ ↑ CD127dim
Kuon *et al*., 2017	After LH peak (7-10 days)	58 RAR group	Fertile women	34.7 years	-	↑ CD56+
Jiang *et al*., 2017	After LH peak (7-9 days)	32 RIF group	Fertile women	32 years	-	↑ CD57+;↓ CD45+ ↓ CD56+↓ FoxP3+
Fukui *et al*., 2017	-	34 RIF group 28 RAR group	Fertile women	RIF (35.3 years) RAR (33.8 years)	↓ TNF-α; ↓ INF-γ	↓ NKp46
Chen *et al*., 2017	After LH peak (7th day)	34 RIF group 97 RAR group	Fertile women	RIF (35 years) RAR (37 years)	-	↑ CD56+
Chen *et al*., 2018	After LH peak (7th day)	18 RAR group	Fertile women	34 years	-	↑ CD56+
Donoghue *et al*., 2019	After LH peak (6-8 days)	14 RIF group 9 PRIF group	Implantation success	RIF (37.4 years) PRIF (38.1 years)	-	No significant differences
Amjadi *et al*., 2020	After LH peak (6-7 days)	11 RIF group	Fertile women	32.6 years	↑IL-2;↑ IL-6 ↑IFN-↑;↑IL-17A ↑IL-23A; ↑ IFN-A1 ↑ IFN-B1	-
Zhao *et al*., 2020	After LH peak (7th day)	30 RAR group	Fertile women	20 - 42 years	-	↑ CD3+;↑ CD56+ ↑ CD68+
Sauerbrunn-Cutler *et al*., 2021	After LH peak (9th day)	17 non-pregnant group	Pregnant women	34.3 years	-	↑ CD16+,↓ FoxP3+
Zhao *et al*., 2021	After LH peak (7th day)	44 non-pregnant group	Pregnant women	35.92 years	-	↑ CD56+;↑ CD68+

CD: Cluster of Differentiation; RIF: Recurrent Implantation Failure; RAR: Recurrent Abortion Rates; LH: Luteinizing Hormone; Th: Helper T Cells; NKp: Natural Cytotoxicity Receptor Protein; and - : not available.

CD: Cluster of Differentiation; RIF: Recurrent Implantation Failures; PRIF: Potential Recurrent Implantation Failures; RAR: Recurrent Abortion Rates; ET: Embryo transfer; LH: Luteinizing Hormone; NKp: Natural Cytotoxicity Receptor Protein; FoxP3+: Forkhead box P3 protein; and -- : not available.

### Common Points Between Collections

**Figure 2:**
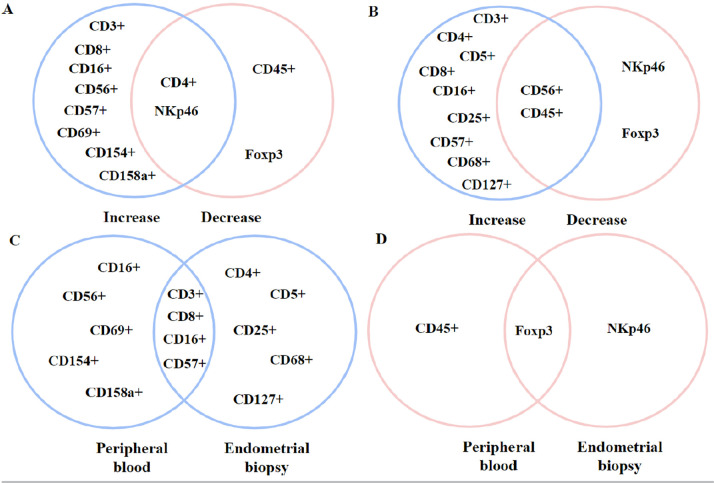
Venn diagram containing peripheral blood immune cells and embryo biopsy in RIF, RAR, and infertile patients. Caption: Immune cells from different types of collection. In blue: increase in cell quantity. In pink: Reduction of cell quantity. A: Peripheral blood immune cells. B: Immune cells derived from embryonic biopsies. C: Just enlarged cells between the two collection types. D: Just reduced cells between the two collection types.

**Figure 3:**
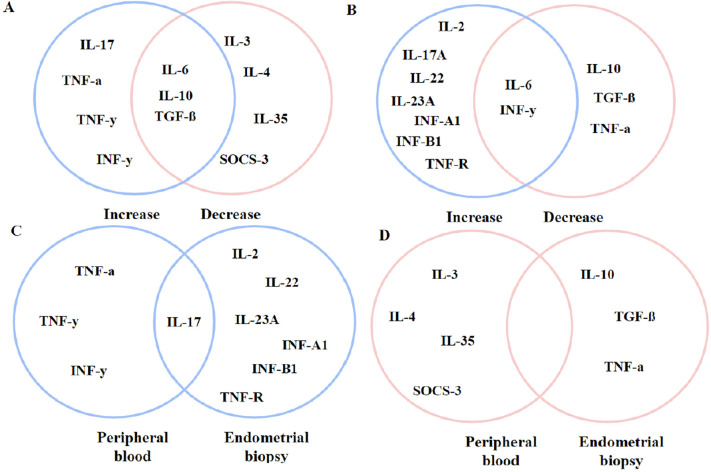
Venn diagram containing peripheral blood inflammatory cytokines and endometrial biopsies from RIF, RAR and infertile patients. Caption: Cytokines from different types of collection. In blue: increase in the number of cytokines. In pink: Reduction in the number of cytokines. A: peripheral blood immunological cytokines. B: Immune cytokines derived from embryonic biopsies. C: Only amplified cytokine pattern between the two types of collection. D: Only reduced cytokine pattern between the two types of collection.

To better plot, the commonalities between both collections, the main immune cells found in the studies were selected and placed in a Venn diagram, as shown in Figures 2 and 3. By plotting the main immune cells, we noticed that there was a common immune enhancement in both techniques in CD3 +, CD8 +, CD16 +, and CD57 + cells. In the cells found, there was an intersection point of the FoxP3+ phenotype. Interestingly, among the inflammatory cytokines in the endometrial biopsies used in the review, the only intersection point found in the study was the increase in IL-17 levels.

## DISCUSSION

In a successful pregnancy, the body must undergo many physiological processes that act directly on proper endometrial function and dynamic interaction between the endometrium and the blastocyst (Mrozikiewicz *et al*., 2021). This implantation process is somewhat inefficient in human reproduction, since about 25% of conceptual matings generate live births, and 5-15% can be identified clinically (Nowak *et al*., 2017). In this context, investigating biomarkers and clinical trials to determine or prospect possible treatment and/or immune abnormalities in these patients could effectively improve conception rates (Parhizkar *et al*., 2021).

One of the methodologies found in this search was the study of immune components from the peripheral blood of patients. Peripheral blood cell analyses consist mainly of evaluating lymphocytes and monocytes that are enabled in a wide range of assays, from the most straight forward, such as cytotoxicity assessments, to the most complex, such as functional and phenotypic evaluation of immune cells (Navas *et al*., 2019). Among the significant disadvantages of peripheral blood cell analyses is the similarity between immune responses in the body, which can lead to erroneous analysis and interpretation of data. However, non-generic-specific biomarkers can improve tissue specificity (Jogia *et al*., 2021). However, one of the main advantages of peripheral blood collection over other collection types is its better accessibility (Medrone Junior, 2009).

Another well-used methodology for the identification of immunological biomarkers was an endometrial biopsy. The primary purpose of this technique is to obtain a sampling of endometrial material to evaluate lesions of the endometrial lining and investigate other benign pathologies (Nicholls-Dempsey *et al*., 2018). One of the main disadvantages of endometrial biopsy is still the invasiveness of the technique. Although it is less harmful compared to other methodologies, such as laparoscopy, it still requires anesthesia or strong analgesics in some cases (Al-Jefout *et al*., 2007). However, the endometrial tissue structure has many distinct molecules that may contribute to adhesion, cell distribution, transit, and more specific signaling processes involved in implantation disorders (Marron *et al*., 2019).

RIF, RAR, and infertile patients, had higher T lymphocyte counts (CD3+, CD4+, CD8+, CD69+, CD154+) in their peripheral blood and (CD3+, CD4+, CD8+, CD25+, and CD127+) in endometrial biopsies (Yang *et al*., 2010; Chernyshov *et al*., 2014; Dons’koi, 2015; Galgani *et al*., 2015; Wang *et al*., 2017; Zhao *et al*., 2020). The literature has already described that T-type lymphocytes are located in the tissue stroma and glandular epithelium. These cells control the Th1 response involved in immune surveillance, preventing excessive trophoblast invasion and assisting in allograft immune tolerance (Zolfaghari *et al*., 2021). However, the literature also reports that in many cases of maternal anti-fetal rejection and gestational problems, there is an increased infiltration of cytotoxic T-lymphocytes into the fetal tissues (Kim *et al*., 2015). This fact was previously observed in the study by Quinn *et al*. (2011) who found an increased infiltration of cytotoxic CD8+ cells in women who had severe late preeclampsia.

Cells of the innate immune response have also been shown to be affected in cases of conception failure. The amount of CD68+ macrophages in endometrial biopsies increased in patients who intended to achieve clinical pregnancy from ART (Zhao *et al*., 2020; 2021). Under normal conditions, peripheral blood macrophages can reduce the invasiveness of trophoblast implantation. However, when these macrophages are dysregulated, there is no complete explanation for their effects on implantation (Han *et al*., 2021). In a previous study by Kim *et al*. (2008) there was an increase of macrophages in chorionic villi of patients with villitis of unknown cause, in addition to the macrophage infiltrate increase in the presence of CD8+ T cells in these patients.

One of the main unknowns in the literature is the impact of NK cells on embryo implantation. The attack of cytotoxic NK cells is immediate and does not require prior antigen preparation. Instead, these cells orchestrate the attack uniquely from interactions with receptors or inhibitory functions (Becker *et al*., 2016). An increase of NK cells in peripheral blood and endometrial biopsies (CD8+, CD16+, CD56+, CD57+, and CD158a+) in most studies (Tuckerman *et al*., 2010; Karami *et al*., 2012; Junovich *et al*., 2013; Chernyshov *et al*., 2014; Dons’koi, 2015; Santillán *et al*., 2015; Jiang *et al*., 2017; Kuon *et al*., 2017; Chen *et al*., 2017; 2018; Sauerbrunn-Cutler *et al*., 2021; Comins-Boo *et al*., 2021; Zhao *et al*., 2021). In a previous retrospective study evaluating the ovarian reserve of women undergoing cycles of ART, they found that the high rate of NK cells led to a reduction in ovarian reserve, which confirms the reproductive impacts shown previously (Hur *et al*., 2020). However, NK cells in the peripheral blood of healthy individuals can vary around 5%-29% depending on gender, stress, ethnicity, and age. Based on this, confirming their actual activity on fecundity it is still a controversial issue in the literature (Canella *et al*., 2021).

A curious finding was that some immune system cells adopted both a growth and a declining pattern in the different studies analyzed. These patterns occurred in dendritic cell lineages (CD86+) in the peripheral blood and NK cells expressing NKp46 receptors. In addition, regulatory T (Foxp3) and (CD4+) cells were also expressed in a lower amount in endometrial biopsies (Jiang *et al*., 2017; Fukui *et al*., 2017; Liu *et al*., 2019; Comins-Boo *et al*., 2021; Sauerbrunn-Cutler *et al*., 2021). As for the lower expression in dendritic cells, the literature already reports that this low amount of cells can lead to a reduction in IL-10 that controls tissue levels of effector T-cells that consequently infiltrate the endometrium (Kong *et al*., 2018). Recent studies have reported that reducing NK cells expressing NKp46 receptors on endometrial cells increases the high risk of gestational loss. However, their correlation with this loss is still unknown in the literature (Fukui *et al*., 2006; Takeyama *et al*., 2021).

Regulatory T-cells are defined by their expression of CD4+, CD25+, and the Foxp3 domain, which control the developmental function of naturally occurring T-reg populations (Heitmann *et al*., 2017). In this review, we found increased CD4+ cells in peripheral blood and a reduction in FoxP3. The primary function of these regulatory cells is endometrium modulation to receive the semi-allograft without complete immunosuppression during implantation, in addition to its protective function against opportunistic infections, which may risk the mother’s health (Royster *et al*., 2019). In a previous study, Teles *et al*. (2013) used mouse models with depletion for regulatory T-lymphocytes and reported that they had potential problems in embryo implantation, which is in tune with the findings of this review.

From the studies analyzed, we found a panel of cytokines sharply increased in the peripheral blood of the patients, including cytokines IL-6, IL-10, IL-17, INF-γ, TGF-ß, TNF-α (Ozkan *et al*., 2014; Saifi *et al*., 2014; Yin *et al*., 2017). However, according to a review by Chaouat *et al*. (2003), after the coitus phase until the apposition phase, the uterus is subjected to an inflammatory reaction with a transient cellular influx of lymphocytes and macrophages near the uterine lumen, leading to a high production of interleukins such as IL-6 and TNF-a, which could explain this change in peripheral blood cytokines, which also showed a reduction in IL-4, IL-6, IL-10, IL-35, FoxP3, TGF-ß, SOCS3 cytokines (Junovich *et al*., 2013; Zhou *et al*., 2012; Ozkan *et al*., 2014; Saifi *et al*., 2014; 2016; Koushaeian *et al*., 2019). On the other hand, when we checked the expression patterns of inflammatory cytokines in endometrial biopsies, IL-2, IL-6, IL-17, IL-22, IL-23, INF-A1, INF-B1, INF-γ, TNF-R were relatively increased in patients with reproductive complications (Galgani *et al*., 2015; Amjadi *et al*., 2020). This fact was reported by Saini *et al*. (2011) saying that Th1- cells, especially IL-2 and gamma interferon producers, play a crucial role in allograft rejection during fetal implantation. On the other hand, the levels of IL-6, IL-10, INF-γ, TGFß, TNF-α had their levels reduced (Junovich *et al*., 2013; Galgani *et al*., 2015; Wang *et al*., 2017; Fukui *et al*., 2017). This fact can also be explained by Chaouat *et al*. (2003) as previously mentioned, in which the reduction of cytokines by defense cells can lead to an unfavorable environment for embryo development, resulting in development imbalance and pregnancy loss. One of the main limitations of that study is the unspecificity of immune factors collected mainly from peripheral blood, as mentioned before, which may account for the increase and decrease in some immune cells (Jogia *et al*., 2021). Another factor that can lead to failures in these methodologies is the collections performed at different times, which can modulate the proliferation of immune cells and make it impossible to collect more homogeneous data and responses. Hormonal balance may be an interesting parameter to be addressed in further studies to investigate its correlation with the immune system and its implantation impacts on ART outcomes (Kim *et al*., 2015).

In conclusion, identifying immunological biomarkers may be an effective way to address fertility issues. Increased cytotoxic T-lymphocytes such as CD3+, CD25+, CD69+, CD127+, and CD154+ have been shown to correlate with fertility in patients. Innate immune response cells CD68+ were also increased in patients with problems to achieve pregnancy. CD8+, CD16+, CD56+, CD57+, and CD158a+ NK cells may exhibit a possible increase in the rates of reproductive problems. However, this finding is still uncertain in the literature. Regulatory T-cells such as Foxp3 have been shown to have the potential to aid in implantation immune tolerance, and their reduction has been seen in patients with reproductive disorders. In this context, further studies are needed to verify these cells’ main activity in the IS and identify functional biomarkers. In addition, the data presented in this review can be of great value for the design of studies aimed at developing methodologies, such as rapid tests that could predict the implantation rate of patients who resort to ARTs, enhancing the chances of clinical pregnancy success.

## Abbreviations

RIF: Recurrent implantation failure
IVF: In vitro fertilization
MESH: Medical Subjects Headings
RAR: Recurrent Abortion Rates
ART: Assisted Reproductive Techniques
PRISMA: Reporting Items for Systematic Reviews and Meta-Analysis and
IS: Immune system
